# Assessing behavioral control across the adult lifespan using a novel outcome revaluation task

**DOI:** 10.1038/s41598-025-29795-5

**Published:** 2025-11-26

**Authors:** Corinna Y. Franco, Barbara J. Knowlton

**Affiliations:** ¹Department of Psychology, University of California, Los Angeles, Los Angeles, CA 90095 USA

**Keywords:** Aging, Habits, Behavioral control, Obsessive-compulsive behavior, Depression, Neuroscience, Psychology, Psychology

## Abstract

While many studies have investigated the effects of aging on cognition, relatively few have examined aging impacts on habitual behavioral control. To assess these relationships, 151 adults across the lifespan (47.09 ± 17.17 years old, range = 19–80) completed a novel instrumental outcome revaluation task, where participants made keyboard responses to abstract stimuli to gain digital currency before completing a revaluation test where the outcome of one stimulus was negatively altered while the other retained its value. Participants also completed questionnaires relating to psychiatric symptoms. Habitual responding was measured in terms of the response rate to the revalued stimulus relative to the response rate to the stimulus that was not revalued. There were significant positive effects of obsessive-compulsive symptoms and significant negative effects of depressive symptoms on habitual behavior. In addition, results revealed a modest effect of chronological age on habitual behavior These results indicate subtle changes in behavioral control across the adult lifespan and support previous work showing that certain psychological measures including obsessive-compulsive symptoms are associated with increased habitual responding.

## Introduction

The inevitability of aging has drawn hosts of questions about the changing neurocognitive landscape of humans across the lifespan^1^. One question that has not yet been fully addressed is whether there are differences in goal-directed vs. habitual control of behavior in older compared to younger adults^2,3^. Evidence of behavioral differences and neuroimaging findings concerning declines in the regions which subserve goal-directed actions point toward a preference for habits in aging, perhaps due to impaired acquisition of goal-directed responding^2–7^.

Of the extant literature, it appears that goal-directed responding suffers impairments while habitual responding remains intact with age. Eppinger et al. (2013)^4^ was one of the first groups to investigate these relationships using a two-stage Markov decision task allowing for the examination of model-based (goal-directed) and model-free (habitual) behavior. Younger adults were able to acquire larger mean payoffs than older adults, indicating potentially greater model-based decision-making benefiting overall task performance in younger groups. Findings from this study pointed to deficits among older adults in updating value representations and creating or updating models of environmental associations critical for beneficial decision-making processes. Older adults also showed a greater tendency to perseverate in responding rather than employing strategic alterations in behavior to maximize reward.

De Wit et al. (2014)^2^ has similarly shown aging deficits in goal-directed, but not habitual, control using an instrumental learning task paired with outcome-devaluation and slips-of-action tests. Results showed that older adults exhibited some difficulty with employing goal-directed behavior to aid in task performance, leading to negative impacts on accuracy, alongside impairments with employing learned Response-Outcome (R-O) associations, compared to young adults. Older adults also had difficulty with mitigating responding to devalued outcomes while maintaining high responding to valuable outcomes across all trial types. Thus, older adults exhibited a deficit in the ability to employ goal-directed over habitual control both during initial training and at test, at times appearing to favor Stimulus-Response (S-R) habitual control overtly.

Finally, Ito et al. (2021)^5^ validated these findings using both in-person and online testing methods through the employment of a two-stage Markov task. Trial-by-trial examinations of young and older adult responding on this task found dual employment of goal-directed and habit control strategies both in-person and online among young adults. Only online-tested older adults showed evidence of the employment of goal-directed strategies, with in-person older adults showing characteristics of a habit-based response strategy. Further examinations indicated that, for both testing formats, younger adults exhibited greater model-based, goal-directed responding than older adults. Greater age also predicted a higher number of perseverative and stochastic responses in this task.

The extant literature provides evidence of preserved habitual response strategies alongside deficits in goal-directed responding in older adults. However, these studies have not shown increases in habitual responding with age that one would expect might occur alongside deficits in goal-directed responding. There are a few reasons why previous research has not found increased habitual behavior in older adults. First, these accounts have employed extreme age group designs, which do not include a large subsection of the adult human lifespan. A lifespan approach may be more sensitive to behavioral change in that it examines aging as a continuous variable. Second, previous designs often did not control for psychological and demographic variables that may impact performance and could differ by age. Third, the tasks traditionally used to examine human behavioral control involve the use of instructed devaluation (participants are simply told that one stimulus will no longer be rewarded) and may not be particularly engaging. In the present study we used a novel behavioral control paradigm in the form of an outcome revaluation task sampling across the adult lifespan that allowed us to effectively assess behavioral control alterations in aging. In the revaluation phase, participants will experience negative consequences for choosing the revalued stimulus and then will be tested for responding to the valued and revalued stimuli during an extinction phase with no outcomes.

For aging populations, who are already vulnerable due to other neurocognitive and physiological health issues, susceptibility to habitual control of behavior can lead to decreases in quality of life, including predispositions toward developing inflexible maladaptive behaviors such as substance use disorders^8^. However, it may also be leveraged toward the creation of safer, healthier habits^9^. Thus, characterizing such behaviors in aging will allow us to determine what aspects of behavioral control are preserved to better understand cognitive decline and other quality of life issues in older adult populations.

The novel outcome revaluation task is modeled after traditional instrumental outcome devaluation paradigms, where participants view stimuli and make associated key presses to receive particular outcomes during an instrumental training phase, after which one stimulus becomes devalued and subsequent responding alterations are assessed. Continued responding to the devalued stimulus is considered a measure of habitual responding^10^. The novel revaluation task makes the following key changes: the devaluation phase is replaced with a revaluation phase, wherein the “devalued” stimulus outcome is altered rather than eliminated; the revaluation phase is exploratory rather than instructed, as often occurs in real-world associative environmental changes; participants make a sequence of responses rather than the traditional singular response during all phases of the task, allowing for a better examination of behavioral control response phenotypes; and the task is constructed to mimic a game-like environment to increase motivation and attention. In the cover story for the task, participants are to imagine that they are chefs making different dishes for a payout, and they believe they are competing against a rival which increased engagement with the task.

Based on previous studies^2,4,5^ we predicted that aging will lead to increased habitual responding and negatively affect goal-directed responding even when controlling for relevant covariates including early life stress exposure, depression, state and trait anxiety, subjective socioeconomic status, current stress, and obsessive-compulsive behavior. Previous work has shown a relationship between increased habitual responding in people who experienced early-life stress^11,12^ and in patients with obsessive-compulsive disorder (OCD;^13–16^. Thus, we wanted to use a multiple regression approach to examine effects of aging independently of these other factors.

Moreover, we hypothesized that aging may have an overall negative effect on instrumental learning accuracy given the literature on impaired reward learning in older adults (e.g^17,18^. Finally, we hypothesized that responding among participants showing habitual behavior would be faster than responding among goal-directed participants across the lifespan based on theoretical perspectives that habits are more automatic than goal-directed actions (e.g^19^.

## Methods

### Participants

Two hundred participants (47.09 ± 17.17 years old, range = 19–80) were recruited online using Prolific (http://www.prolific.com). Using G-power, the necessary sample size was determined a priori to be 152 based on a 0.90 probability of obtaining a moderate effect (Cohen’s f = 0.015) with 11 predictors. We oversampled given our pilot data with similar online tasks for which there was a 10–25% participant exclusion rate due to performance issues. In the present study, 49 participants (51.35 ± 16.10, range = 19–76) were excluded due to poor learning (learning accuracy < 50%; *n* = 24), test performance (responding to valued stimuli at test < 4; *n* = 9), and incorrect selection of the revalued stimulus (see Design and Procedure; *n* = 16). Although we did not screen participants for age-related cognitive decline, our exclusion based on performance measures would have eliminated participants who did not understand task instructions or were distracted. The final sample consisted of 151 participants (45.72 ± 17.32 years old, range = 19–80, 80 female; 71 participants were 40 and younger, 54 participants were between 41 and 65, and 26 were older than 65; see Table 1 for demographic information).

**Table 1:** Participant demographics and psychological measures.

Demographics	Age Mean (SD)	BDI Mean (SD)	STAI Y1 Mean (SD)	STAI Y2 Mean (SD)	PSS Mean (SD)	SSES Mean (SD)	OCI-*R* Mean (SD)	Overall early life stress Mean (SD)
Gender								
Female (*n* = 80)	45.35 (17.22)	14.91 (12.75)	37.14 (14.50)	43.12 (14.57)	17.90 (8.93)	4.92 (1.56)	33.92 (14.63)	67.35 (8.65)
Male (*n* = 71)	46.13 (17.55)	10.99 (11.53)	34.49 (13.18)	38.61 (13.46)	14.61 (8.33)	5.08 (1.88)	34.62 (16.32)	52.63 (17.33)

Participants provided informed consent and were compensated US $12 per hour of participation. The Institutional Review Board of the UCLA Human Research Protection Program granted approval for all study procedures. All procedures were conducted in accordance with the Declaration of Helsinki, and all participants provided informed consent before participating in the study,

### Design and procedure

Participants completed the experiment on their own computing devices. Participants were told that the aim of the study was to examine the effects of aging and stress on non-declarative learning and that they will be asked to do an online task where they will make different keyboard presses in response to distinct visual stimuli. They were also told that after completing the task, they will be asked a series of questions relating to demographic characteristics, early life stressors (e.g. childhood trauma and abuse), mental health (e.g. depression, anxiety), substance use, and other health behaviors, and that the entire session would last one hour. The behavioral task was created using PsychoPy (version 2023.2.2, http://www.psychopy.org; Peirce et al., 2019) while questionnaire responses were formatted and collected via Qualtrics (Provo, UT; http://www.qualtrics.com). The task took participants approximately 22 min and they spent the next 20–30 min completing questionnaires. There were no enforced breaks during the task, but participants could read the instructions for the different task phases and could complete the questionnaires at their own pace. Participants received compensation for their participation.

**Fig. 1:**
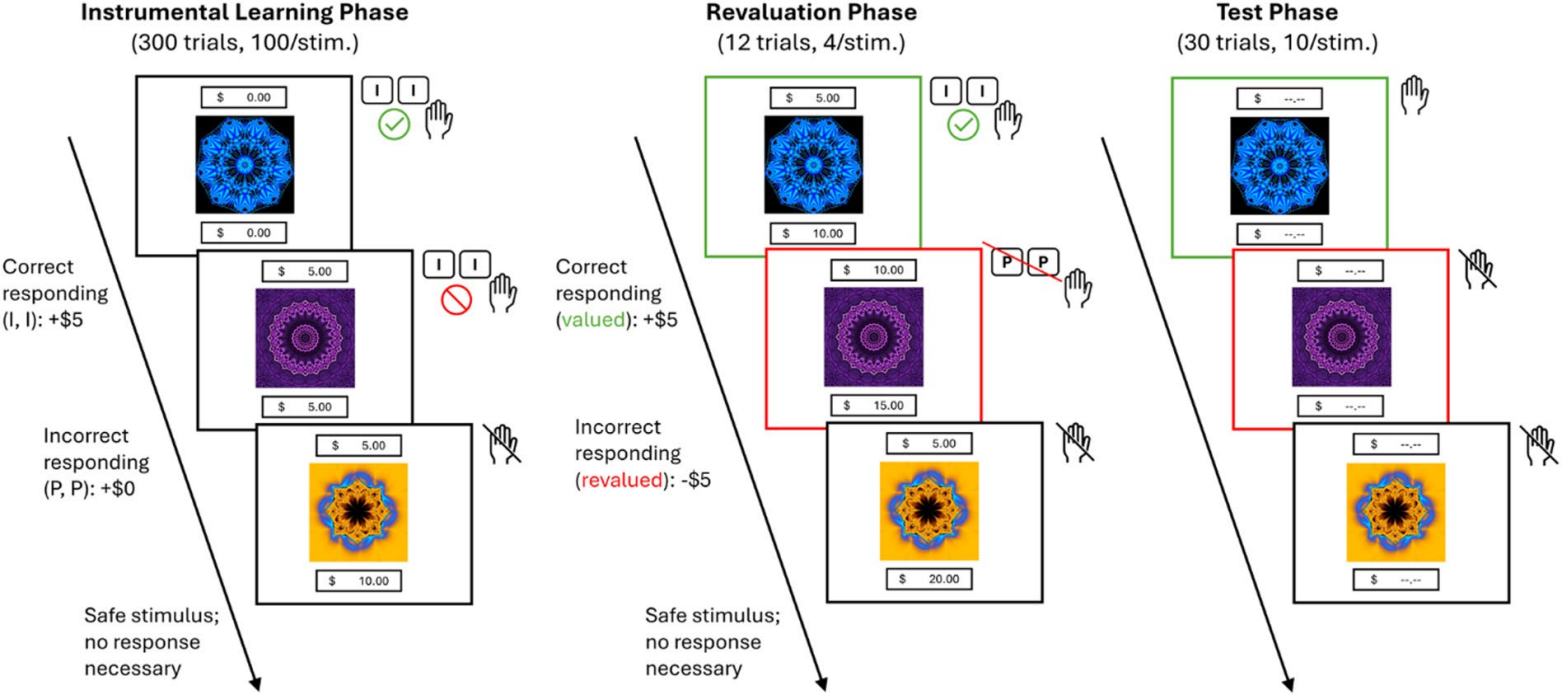
Schematic of the appetitive outcome revaluation task. Participants were tasked with viewing abstract stimuli and making an associated response (II or PP) to gain currency (+$5.00) and beat a virtual rival (300 trials, 100 per abstract stimulus; participant counter – top, rival counter – bottom). Following an initial instrumental learning phase, one of these stimuli was revalued, now associated with monetary loss (-US $5.00) instead of gain (+ US $5.00). Participants had to figure out which stimulus was revalued on their own (12 trials, 4 per stimulus). Participants then completed a test phase under these revalued conditions, where they were told to keep responding to the unaltered stimulus but not the revalued stimulus, while their currency counters were broken (30 trials, 10 per abstract stimulus; 342 trials total).

To measure behavioral control, participants completed the novel appetitive outcome revaluation paradigm. Participants were told that they would act as a “chef” and they had to make one of three dishes corresponding to the stimulus presented on the screen by pressing the correct keypress sequence. The task was composed of three phases: an instrumental learning phase, a revaluation phase, and a test phase (see Fig. 1).

During the instrumental learning phase, participants were shown a randomized set of 300 trials of three distinct, sequentially presented abstract stimuli (2-s. duration, 500 ms ITI, 100 trials/stimulus). Two of these stimuli required responses to gain points (active), while the third stimulus acted as a neutral “safe” stimulus wherein responding did not lead to gain or loss (passive). Participants were tasked with making the correct keyboard response (either II or PP) to make the correct dishes corresponding to the active stimuli to gain “money”, with US $5.00 acquired for each correct response, noted in a virtual currency counter at the top of their screen. Note that responding involved two key presses per stimulus to examine how individuals altered responding to stimuli when a series of actions, not simply one action as is common, needed to be completed. Participants had to compete against a digital “rival chef” to earn as much money as possible to enhance task motivation. The rival’s virtual currency counter was present at the bottom of their screen and was randomly incremented throughout the task.

Following this learning phase, participants underwent a revaluation phase wherein one of the active stimuli became revalued (12 trials total, 4 trials/stimulus). Participants were told that the machine to make one of the dishes was broken and they would lose money if they tried to make one of the dishes. Participants had to keep responding to the unaltered stimulus to earn money (+ US $5.00), but responding to the revalued stimulus now led to a penalty, depicted as a loss of currency (-US $5.00) in their virtual counter. Participants needed to determine which stimulus was revalued on their own through examination of currency accrual or loss when responding to the stimuli. Following completion of this phase, participants were asked which stimulus they believed was revalued. After the revaluation phase, participants underwent a further testing phase (10 trials/stimulus, 30 trials total), which acted similarly to the revaluation phase save for the elimination of the point counters informing stimulus-response-outcome learning. Participants were told that they should continue with the task but they will not receive feedback about their earnings. Continued responding to the revalued stimulus during the test phase was assessed as habitual behavior, as these responses were insensitive to outcomes given that they were now associated with negative consequences instead of positive ones.

After finishing the behavioral task, participants completed a series of questionnaires to acquire covariates known to influence behavioral control. These covariates included early life stress (childhood trauma and abuse) exposure, depression, state and trait anxiety, subjective socioeconomic status, current stress, and obsessive-compulsive behavior which were measured via the Childhood Trauma Questionnaire Short Form (CTQ-SF;^20^, the Beck Depression Inventory (BDI;^21^, the State and Trait Anxiety Forms Y1 and Y2 (STAI Forms Y1 & Y2;^22^, the MacArthur Scale of Subjective Social Status (SSES;^23^, the Perceived Stress Scale (PSS;^24^, and the Obsessive-Compulsive Inventory-Revised (OCI-R;^25^, respectively.

## Data analysis

Data were analyzed using R (version 4.4.3, http://www.r-project.org; R Core Team, 2025). We used the psych (version 2.4.12, CRAN.R-project.org/package = psych;^26^, emmeans (version 1.10.7, CRAN.R-project.org/package = emmeans;^27^, visreg (version 2.7.0, pbreheny.github.io/visreg;^28^, and ggplot2 (version 3.5.1, ggplot2.tidyverse.org;^29^ packages for descriptive statistics, post-hoc analysis, and data visualization, respectively. Sex was dummy coded in all relevant analyses (0 = male, 1 = female). All continuous predictors were mean centered in relevant analyses. A significance level of 0.05 was utilized for all analyses.

Habitual responding was derived as the number of “correct” (associated) revalued stimulus responses on a scale of 0 (no habit responses) to 10 (out of 10 possible habit responses) completed during the test phase. Instrumental learning accuracy (percent correct responses during the learning phase) for the active stimuli and correct responding to the unaltered stimuli during revaluation and at test were also acquired. Additionally, reaction times for every correct stimulus response (all stimuli during learning, revalued and unaltered at test) were collected and averaged for each participant. A reaction time subtraction measure was calculated as the reaction time for the second response subtracted from the reaction time to the first response in order to examine the speed at which both responses were made in relation to one another (e.g. second I response – first I response). Early life stress exposure, depression, state/trait anxiety, current stress, subjective socioeconomic status, and obsessive-compulsive symptoms were used as covariates in all analyses unless otherwise stated.

Where applicable, results were visualized using partial regression plots, which allow for the illustration of the relationship between a predictor and criterion while controlling for other variables in regression models.

## Results

We first characterized learning and behavioral control outcomes to ascertain the validity of the novel outcome revaluation task. Average instrumental learning accuracy was 91.94% ± 10.67%, signifying high stimulus-response-outcome learning despite added response complexity. Participants made an average of 2.40 ± 3.89 responses to the revalued stimuli at test, ranging from 0 to 10, indicating high variability in habit responding among our sample. Participants also made an average of 9.41 ± 1.10 responses to the unaltered stimuli at test, indicating understanding of task instructions. Mean reaction times are shown in Table 2.

**Table 2:** Reaction times across and within phases.

Task phase	Reaction time Mean (SD)	Reaction time Median
Overall phase RTs		
Learning phase	770 (140) ms	740 ms
Test phase	710 (160) ms	670 ms

Stimulus valuation	Reaction time Mean (SD)	Reaction time Median
Test phase RTs		
Unaltered	710 (160) ms	670 ms
Revalued	880 (200) ms	860 ms
Initiation of responses at test		
Unaltered	600 (150) ms	560 ms
Revalued	760 (190) ms	740 ms

To characterize relationships between age and the model covariates, linear regression analyses were conducted examining age effects on depression, state/trait anxiety, current stress, subjective socioeconomic status, obsessive-compulsive symptoms, and early life stress exposure. Results showed negative effects of age on depression (b = -0.23, *p* < 0.001, 95% CI [-0.34, -0.13]), state anxiety (b = -0.30, *p* < 0.001, 95% CI [-0.420, -0.18]), trait anxiety (b = 0.33, *p* < 0.001, 95% CI [-0.45, -0.21]), current stress (b = -0.19, *p* < 0.001, 95% CI [-0.26, 0.11]), and obsessive-compulsive behavior (b = -0.26, *p* < 0.001, 95% CI [-0.40, -0.120]) and a positive effect of age on subjective socioeconomic status (b = 0.017, *p* = 0.031, 95% CI [-0.084, 0.11]). Age had no significant effect on early life stress exposure (*p* = 0.82), These results indicate that increasing age is associated with more positive psychological and demographic profiles, including lower depression, anxiety, stress, and obsessive-compulsive symptoms and higher self-perceptions of socioeconomic status. Older adults appear to report similar rates of early life stress exposure, as well, demonstrating similar and stable subjective experiences of childhood trauma exposures.

**Fig. 2:**
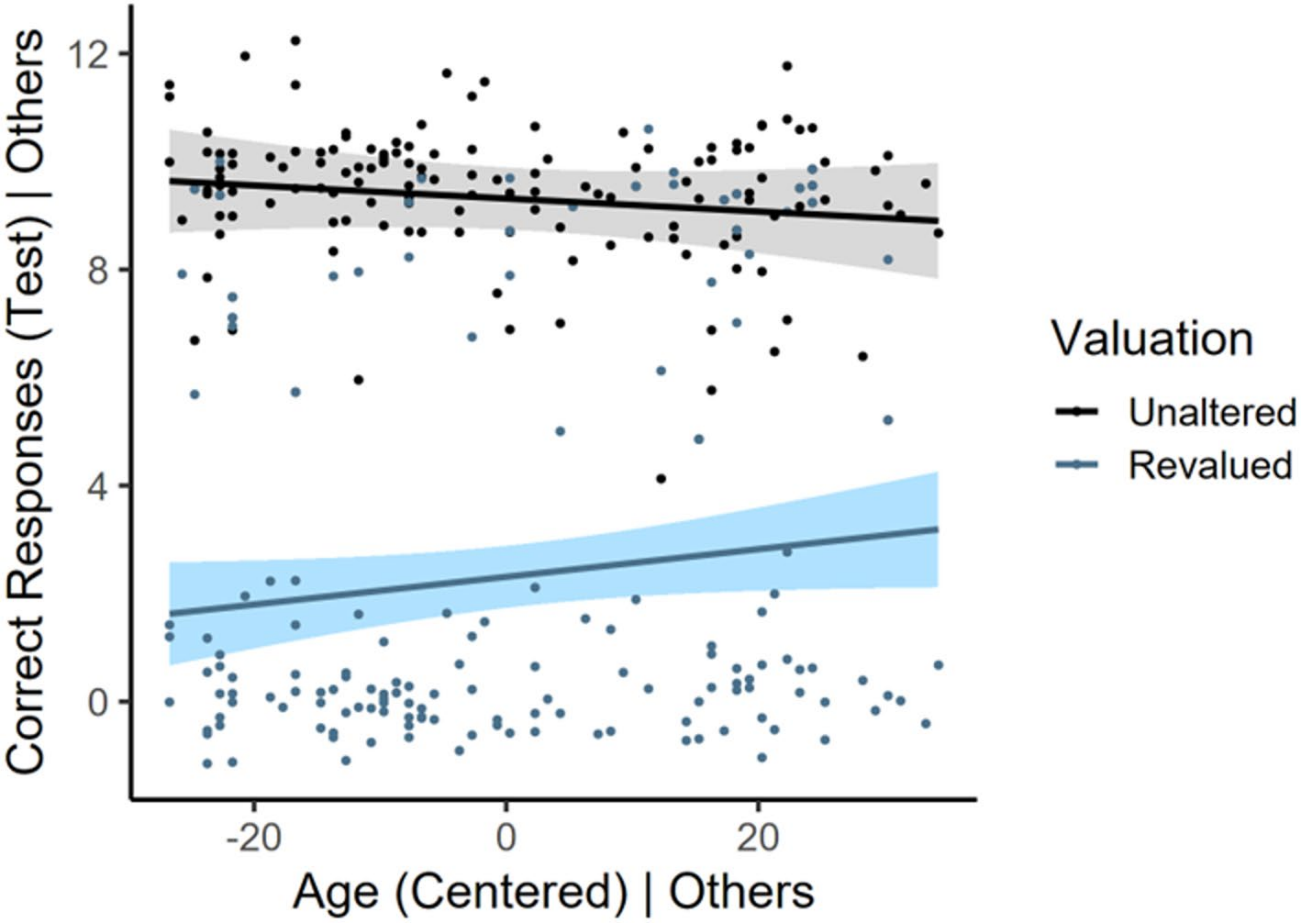
Partial regression plot depicting age effects on correct responding at test. Despite a significant age x stimulus valuation interaction, simple slopes were not significant, though the effect of age on revalued stimulus responding approached significance (top). Shaded regions = 95% CIs. The ‘| Others’ terminology clarifies that these graphs partial out the variance of the covariates in the referenced model, thus residuals are shown.

To assess age effects on behavioral control, we conducted two multiple linear regression analyses using the number of correct responses at test as the outcome and age, stimulus valuation (unaltered, revalued), and age x stimulus valuation as the predictors. The covariates noted in the Data Analysis section were included in the model. Results for the correct responses at test model showed a significant effect of stimulus valuation (b = -7.007, *p* < 0.001, 95% CIs [-7.64, -6.38]) qualified by a significant age x stimulus valuation interaction (b = 0.038, *p* = 0.043 95% CIs [0.001, 0.074]). A simple slopes analysis did not show significant effects of age on number of test responses for the unaltered stimulus (b = -0.012, *p* = 0.39) nor the revalued responses (b = 0.026, *p* = 0.066), though the latter approached significance. Thus, while participants responded more to unaltered stimuli (+ 7.007 responses on average compared to revalued stimuli), controlling for all other variables at their means, age was associated with a change in the pattern of responding to the revalued and unaltered stimuli, consistent with an increased tendency toward habitual responding (see Fig. 2).

**Fig. 3:**
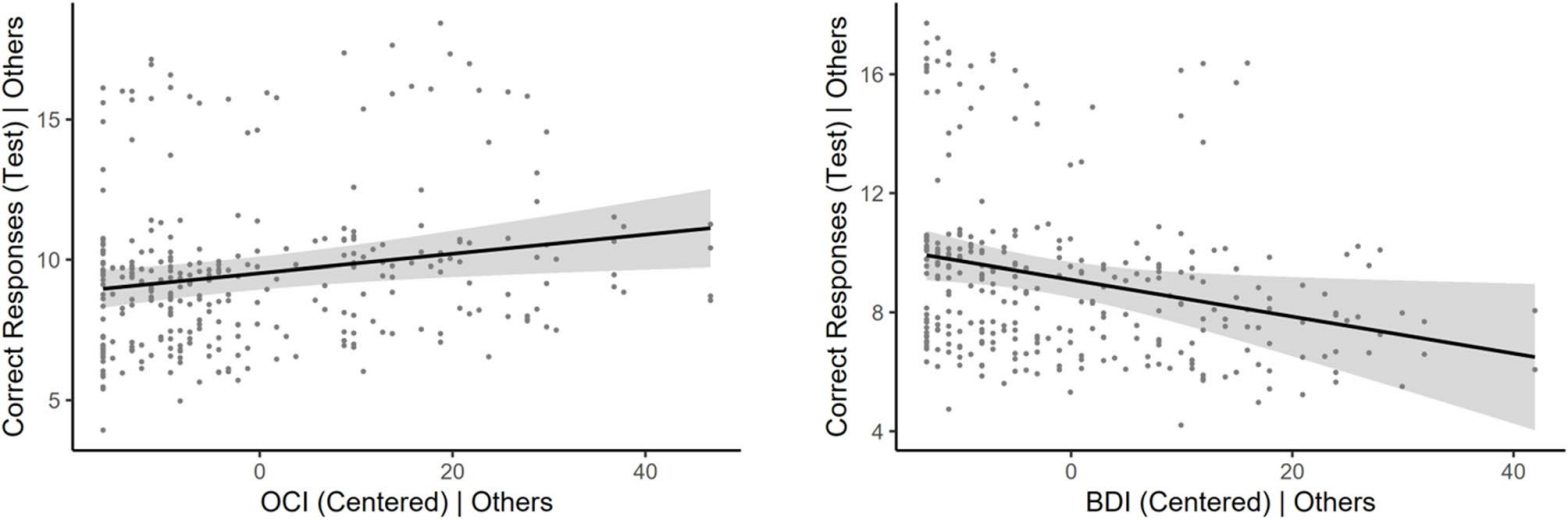
Partial regression plots depicting obsessive-compulsive and depressive effects on correct responding at test. Obsessive compulsive symptoms had a significant positive effect (left) while depressive symptoms had a significant negative effect on correct responses at test (right). Shaded regions = 95% CIs. The ‘| Others’ terminology clarifies that these graphs partial out the variance of the covariates in the referenced model, thus residuals are shown.

A significant positive effect of obsessive-compulsive symptoms (b = 0.034, *p* = 0.009 95% CIs [0.009, 0.060]) and a significant negative effect of depressive symptoms (b = -0.062, *p* = 0.025 95% CIs [-0.12, -0.008]) were found indicating that among participants average on all other terms, each unit increase in obsessive-compulsive symptoms predicted 0.034 more habitual responses, while each unit increase in depressive symptoms predicted 0.062 fewer habitual responses (see Fig. 3). All other terms were not significant (*ps* > 0.11).

Results from two multiple regression analyses predicting revalued and unaltered stimulus responses in isolation clarified that obsessive-compulsive symptoms (b = 0.072, *p* = 0.003, 95% CIs [0.025, 0.12]) and depressive symptoms (b = − 0.12, *p* = 0.025 95% CIs [− 0.22, − 0.015]) predicted revalued stimulus responses, but not unaltered stimulus responses (obsessive-compulsive symptoms: *p* = 0.55, 95% CIs [− 0.018, 0.010] depressive symptoms: *p* = 0.59, 95% CIs [− 0.038, 0.022]). Sex did not predict any behavioral measures except responding to the valued stimulus at test, which was greater in men (b = 0.45, *p* = 0.015, 95% CIs [0.090, 0.81]).

**Fig. 4:**
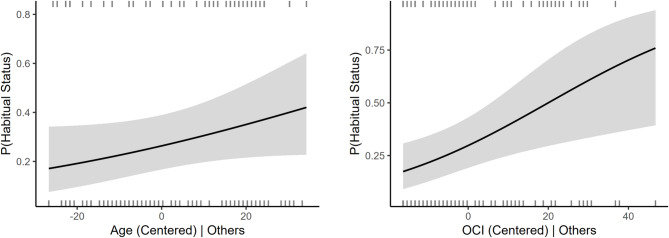
Adjusted probability plots of habitual status (non-habitual, habitual) by age and obsessive-compulsive symptoms. While age did not significantly predict habitual status (left), obsessive-compulsive symptoms was a significant positive predictor (right), such that for each unit increase in obsessive-compulsive symptoms, the odds of responding habitually increase by 4.4%. Shaded regions = Wald 95% CIs. Tick marks represent observations for positive (top, y = 1) and negative (bottom, y = 0) residuals.

Next, we explored whether binomial characterization of participants’ revalued responses (those with and without habit responses during the test phase) better represented our data. We performed a multiple binary logistic regression analysis using a dichotomized representation of participant responding to the revalued stimulus, termed habitual status, as the outcome. That is, participants who performed any revalued stimulus responses were deemed habitual (1) while those who did not were deemed non-habitual (0). Age was the predictor, and we included the relevant covariates (see Fig. 4).

Similar to the previous linear regression analyses, results showed only a trend for an effect of age (*p* = 0.084), but did show a significant positive effect of obsessive-compulsive symptoms (b = 0.043, SE = 0.016, *p* = 0.006), indicating that each additional unit of obsessive-compulsive symptoms was associated with 1.044 times the odds of responding to the revalued stimulus (OR = 1.044, 95% CI [1.013, 1.078]) or a 4.4% increase in odds of revalued stimulus responding. No other effects were significant (*ps* > 0.084).

**Fig. 5:**
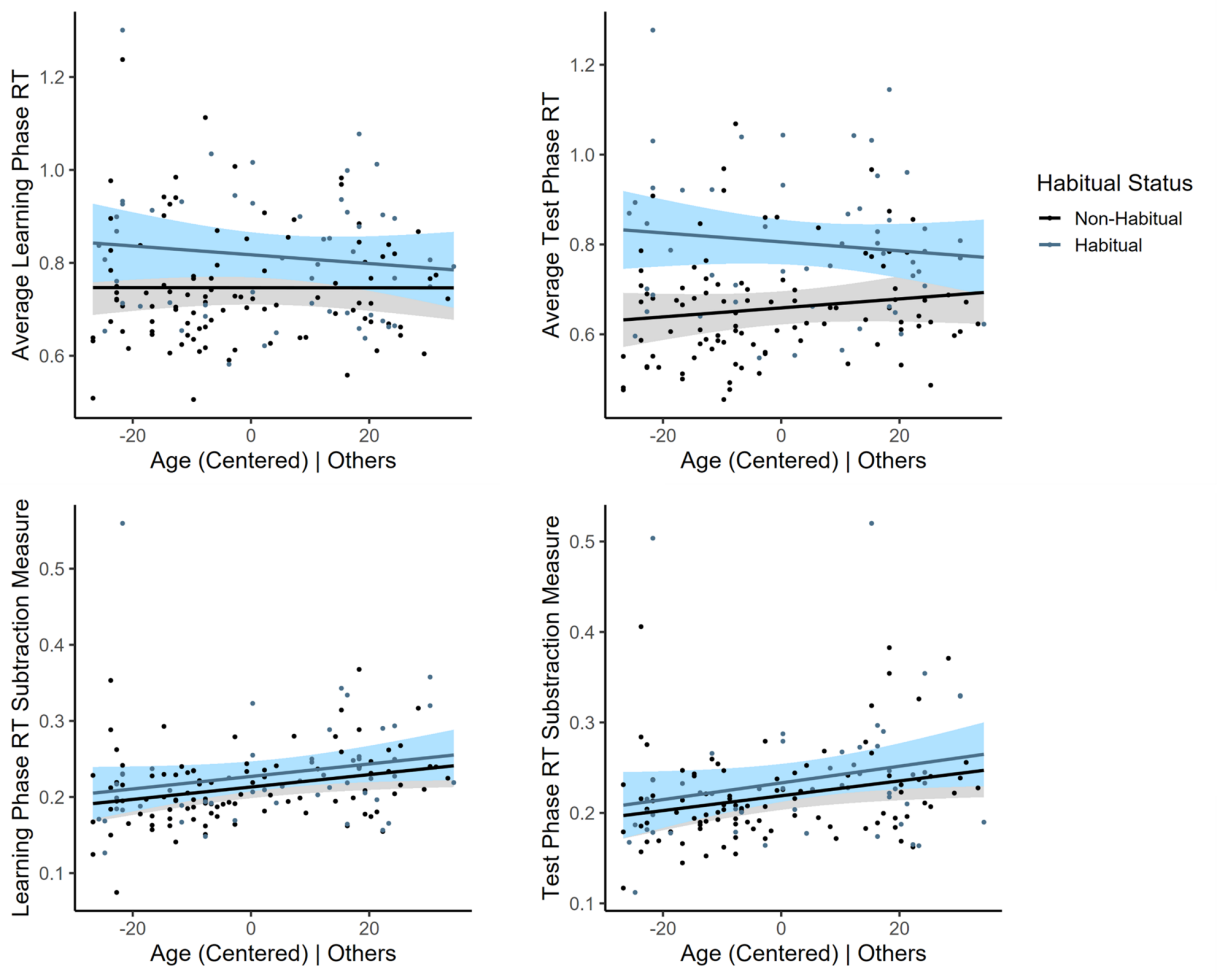
Partial regression plots depicting effects of age and habitual status on various reaction time measures. Habitual status, but not age, significantly predicted average learning and test phase reaction times (sec.), with habitual participants responding significantly slower than non-habitual participants. Age, but not habitual status, significantly predicted the learning and test phase subtraction measures, indicating that older adults had larger gaps between the first and second responses than younger adults. Shaded regions = 95% CIs. The ‘| Others’ terminology clarifies that these graphs partial out the variance of the covariates in the referenced model, thus residuals are shown.

We hypothesized that age-related effects would be observed in reaction times to stimuli both during learning and at test, as well as in the reaction time subtraction measure. As such, four linear regression analyses were performed, using average learning phase reaction time, average test phase reaction time, the learning phase reaction time subtraction measure, and the test phase reaction time subtraction measure as the outcomes; age, habitual status (non-habitual, habitual), and an age x habitual status interaction term as the predictors; and using the relevant covariates (see Fig. 5).

Results for the learning phase reaction time and test phase reaction time analyses found only effects of habitual status, such that individuals who were habitual were unexpectedly slower than individuals who were non-habitual controlling for all other terms (learning phase model: b = 0.071, *p* = 0.005, 95% CI [0.022, 0.120]; test phase model: b = 0.15, *p* < 0.001, 95% CI [0.096, 0.20]). All other terms were not significant (learning phase model: *ps* > 0.17; test phase model: *ps* > 0.16). For the subtraction models, only effects of age were found, such that controlling for all other terms, greater age predicted larger gaps between responses during both the learning and test phases (learning phase model: b = 0.0008, *p* = 0.023, 95% CI [0.0001, 0.002]; test phase model: b = 0.0008, *p* = 0.031, 95% CI [0.000077, 0.002]). No other terms were significant (learning phase model: *ps* > 0.17; test phase model: *ps* > 0.18).

To examine whether there were effects of age on initial instrumental learning, a multiple linear regression analysis was conducted with proportion instrumental learning accuracy as the outcome, age as the predictor, and with all covariates included. Results showed no significant effects of any term on accuracy (*ps* > 0.076), including age (*p* = 0.49). As such, we can determine that adults across the lifespan are able to learn instrumental actions at similar rates.

## Discussion

The current study aimed to examine effects of lifespan aging on behavioral control, including responding and reaction time phenotypes, and determine if aging had a negative impact on instrumental learning using a novel outcome revaluation task. Our findings were generally consistent with our predictions that aging would be associated with greater evidence of habitual behavior. Responses during the test phase were predicted by an interaction of age and whether the stimulus was revalued or not, consistent with an age-related increase in habitual responding relative to overall responding. Lifespan aging did not significantly predict responding to the unaltered stimulus at test, but there was a trend for aging to predict responses to the revalued stimulus. A relationship emerged between habitual responding and obsessive-compulsive symptoms and depressive symptoms, with significant positive and negative effects, respectively, on responding at test and to revalued stimuli. Concerning reaction time phenotypes, contrary to our predictions, individuals who made habitual responses were significantly slower on average during the instrumental learning and test phases than their non-habitual counterparts, and this effect did not interact with age. In fact, age had no effect on average reaction times during these phases. However, older adults did appear to be slower in making their second key press response inputs across all stimuli than younger adults, but this effect also did not interact with tendency to behave habitually.

These findings contrast somewhat with studies which show similar habitual responding tendencies in older adults as in young adults and reduced goal-directed actions^2–5^. Our use of a lifespan design with age as a continuous variable may have been more sensitive to differences in habit performance than designs comparing younger and older participant groups. In addition, our use of a brief revaluation phase, where participants experienced the negative outcome of responding to the revalued stimulus, rather than only instructing participants about the outcome devaluation, may have helped differentiate habitual and goal-directed responders.

In the present results, there were no significant age-related effects on instrumental learning or responding to the valued stimulus during test, suggesting that goal-directed learning was not significantly impacted in our task. This intact level of performance on our instrumental learning task contrasts with the extensive literature showing negative aging effects or differences in reward learning, model-based learning, and associative learning, which underlie the processes necessary to learn stimulus-response-outcome or action-outcome associations integral to instrumental actions^17,30–34^. In the present experiment, the task was designed to be engaging, incorporating a virtual “rival”. In addition, the task was performed online with the participant’s own device so older adults may have felt less anxiety than in the laboratory. However, our results do coincide with some direct studies of aging-instrumental action relationships, which show that older adults learn action-outcome contingencies and instrumental actions as well as their younger counterparts^3,35^. In our study, there was a numerical negative effect of age on responding to the valued stimulus at test, though it did not approach significance. It is possible that aging effects on goal-directed action emerge with more difficult model-based learning paradigms.

The revaluation aspect of the task required participants to switch responding based on an updated contingency. There is substantial evidence for age-related declines in cognitive flexibility as measured by task-switching paradigms. In particular, older adults have difficulty compared to younger adults in mixed task blocks when multiple task sets must be held in mind and task sets other than the current one must be inhibited^36–39^. Likewise, in the present study, after the revaluation phase, participants would need to inhibit the originally learned response to the revalued stimulus. We believe that reduced cognitive flexibility and overreliance on habit are closely related constructs. Stronger habits would be more difficult to inhibit after revaluation and difficulty maintaining competing task sets could result in habitual responding that is not resource demanding. Future work using different paradigms could help integrate these constructs.

Other known influences on behavioral control were found to predict habitual responding in this novel task, namely obsessive-compulsive symptoms and depressive symptoms. Concerning the former, researchers have previously found that patients with OCD exhibit greater habit behaviors than individuals without OCD^13–16^. Our results support this account, providing increased evidence that greater obsessive-compulsive symptomology leads to a preponderance of appetitive habitual responding. In our study, about a third (50/151) participants reported obsessive-compulsive symptomatology levels indicating possible clinical OCD. However, the majority of participants reported subclinical levels. Our finding showing a negative predictive effect of depressive symptoms on habitual behavior, however, was unexpected. Depression has been associated with dysfunctional reward processing and reduced reward responsiveness^40–43^. Again, about a third of participants (50/151) reported depressive symptoms indicating possible clinical levels of depression. Depression has also been shown to relate to decreased devaluation sensitivity and an increase in model-free (habit) behaviors, usually via some mediating factor^44–46^. Thus, one would have expected increased habitual behavior and decreased instrumental learning accuracy among greater depressive individuals, but we observed neither. It is possible that continued responding following revaluation conditions may be more effortful and thus less motivating to individuals with greater depressive symptoms, leading to decreases in habit formation. Another explanation may be that depression-related hyper/differential sensitivity to punishment, as has been shown in some studies^47,48^, led participants with higher depressive symptoms to appropriately terminate responding to the revalued stimulus, now associated with a monetary penalty. Regardless, these results provide evidence that our novel instrumental outcome revaluation task can effectively measure behavioral control and may be a viable alternative than other behavioral control tasks.

Concerning our reaction time analyses, we also found effects contrary to our predictions. When responding is governed by habitual control, the initiation of responses is thought to occur quickly. However, that was not the case in our data, where we observed slower reaction times among habitual individuals during both learning and test. This may be due to the relatively large window of responding allowed for each trial in our task (2 s.), which was chosen due to demographic and task considerations. Namely, older adults are shown to have slower cognitive processing and reaction speeds than younger adults^49,50^, as seen in our subtraction reaction time measures, so shorter response windows likely would have led to higher errors of omission with increasing age. Additionally, other habit tasks have only required the use of one response as opposed to two per trial as in this task, requiring a larger time window to ensure ample response time. However, one recent study has shown that the manifestation of a measurable habitual response is likely to occur within a 300–600 ms window, with shorter and larger response windows leading to a decreased or zero chance of generating a habitual response^51^. Despite average initiation of first responses at test in this task occurring within this 300–600 ms time frame (600 ± 150 ms, median = 570 ms), we saw that first responses to habitual stimuli occurred well after this range (760 ± 190 ms, median = 740 ms). While peculiar, this may indicate an error in overwriting the habitual response, such that individuals attempted to process associative alterations and alter their responding to the revalued stimulus but generated the habitual response regardless. It may also indicate that for tasks with added responding complexity, habitual responders may be slower due to added complex processing failure while valued stimuli benefit from the lack of cognitive updating. Regardless, future studies should consider shortening response windows and adding response complexity to task structures to determine how behavioral phenotypes may differ under these conditions.

This study must be evaluated under some key limitations. Primarily, it was limited by its use of online testing. While online testing is convenient, allows for the acquisition of larger, more diverse sample sizes, and has been shown to produce data with similar quality to in-person testing^52,53^, some studies have shown differences in data quality between online and in-person testing^54^. Online testing is less familiar to older adults, who have less experience with computer/internet use than younger adults. Thus, there was likely a cohort effect, as older adults who participate in online studies may be less representative of their age group than younger participants who have more experience with computers. Participants who were able to complete the task successfully exhibited excellent task performance and understanding, as shown by their high instrumental learning accuracy and valued stimulus responding scores. The older adults who successfully completed the online task may tend to be cognitively healthier than their peers which could have reduced age-related impairments. On the other hand, it is possible that our use of a computer-based task using unfamiliar abstract stimuli and response deadlines may have exacerbated the age-related increase in habitual responding. Using ecologically valid tasks and allowing more time to respond can attenuate age-related impairments^55^. It is possible that with more familiar stimuli and self-paced responding, older adults would not show increased habitual responding. Nevertheless, even when controlling for several covariates, age was associated with a modest tendency for an increase in habitual behavior relative to goal-directed responding.

In sum, the present study used a novel instrumental learning task to probe behavioral control across the lifespan. When controlling for several psychological and demographic variables, we found that increasing age was associated with relatively more habitual responding to a stimulus with a revalued outcome. As hypothesized, increasing OCD symptoms were associated with greater habitual responding. There was also an unexpected negative relationship between depression symptoms and habitual responding. The results suggest that behavioral control, that is, the balance between goal-directed actions and habits, may change subtly across the lifespan, and that some subclinical psychological variables may be related to the propensity to develop habits.

## Data Availability

Data will be made available on request to the corresponding author.
